# Substrate stereoselectivity of poly(Asp) hydrolase-1 capable of cleaving β-amide bonds as revealed by investigation of enzymatic hydrolysis of stereoisomeric β-tri(Asp)s

**DOI:** 10.1186/s13568-015-0118-3

**Published:** 2015-06-04

**Authors:** Tomohiro Hiraishi, Hideki Abe, Mizuo Maeda

**Affiliations:** Bioengineering Laboratory, RIKEN, 2-1 Hirosawa, Wako, Saitama 351-0198 Japan; Bioplastic Research Team, Biomass Engineering Program Cooperation Division, RIKEN Center for Sustainable Resource Science (CSRS), 2-1 Hirosawa, Wako, Saitama 351-0198 Japan

**Keywords:** Poly(Asp) (PAA), PAA hydrolase-1, *Pedobacter* sp. KP-2, β-Tri(Asp)s, Stereoselectivity

## Abstract

We previously reported that poly(Asp) hydrolase-1 (PahZ1_KP-2_) from *Pedobacter* sp. KP-2 selectively, but not completely, cleaved the amide bonds between β-Asp units in thermally synthesized poly(Asp) (tPAA). In the present study, the enzymatic hydrolysis of stereoisomeric β-tri(Asp)s by PahZ1_KP-2_ was investigated to clarify the substrate stereoselectivity of PahZ1_KP-2_ in the hydrolysis of tPAA. The results suggest the following structural features of PahZ1_KP-2_ at its substrate binding site: (1) the active site contains four subsites (2, 1, −1, and −2), three of which need to be occupied by Asp units for cleavage to occur; (2) for the hydrolysis to proceed, subsite 1 should be occupied by an l-Asp unit, whereas the other three subsites may accept both l- and d-Asp units; (3) for the two central subsites between which cleavage occurs, the (l-Asp)-(d-Asp) sequence is the most favorable for cleavage.

## Introduction

β-Peptides, in which monomer units are connected at the β-position, fold into a conformationally ordered state in solution to exert their unique properties and potentially show their enzymatic and metabolic stabilities, which are consistent with the advantages of α-peptides such as interactions with proteins, DNA/RNA, and cell membranes (Seebach et al. 2004; Seebach and Gardiner 2008). Accordingly, β-peptides have recently attracted a wide variety of interests as functional materials.

Poly(Asp) (PAA) is a bio-based, biocompatible, biodegradable, and water-soluble polymer that shows high polyanionic properties because of its high content of carboxyl groups relative to its molecular weight (Freeman et al. 1996; Kim et al. 1996; Tang and Wheeler 2001; Joentgen et al. 2003; Thombre and Sarwade 2005). Methods of synthesizing PAA have been studied, particularly the thermal synthesis of PAA (Kim et al. 1996; Ross et al. 2001; Joentgen et al. 2003; Thombre and Sarwade 2005). Thermally synthesized PAA (tPAA) is chemically prepared on a large scale from l-Asp, resulting in low production cost compared with the microbial synthesis of polymers such as poly(malate) and poly(glutamate). From earlier structural analyses, β-Asp units account for 70% of the total composition in tPAA, indicating that tPAA is composed of a high proportion of β-peptides. The analyses also showed that tPAA contains equivalent moles of d- and l-Asp units and branching and cross-linking as unnatural structures in addition to β-Asp units (Pivcova et al. 1981, 1982; Wolk et al. 1994; Matsubara et al. 1998; Nakato et al. 1998).

Owing to the unique structural and functional characteristics of tPAA, tPAA can be used as an environmentally benign material for dispersing agents and scale inhibitors. In addition to its application as a homopolymer, tPAA derivatives, including block copolymers with poly(ethylene glycol) and hydrogel cross-linked via diamine, have attracted attention owing to their potential applications in the medical and industrial fields as drug delivery systems and superabsorbent materials (Thombre and Sarwade 2005; Osada et al. 2009). Accordingly, it is considered that tPAA biodegradability should be carefully taken into consideration in its applications for practical use.

Generally, unnatural structures in polymer materials affect the biodegradability of the materials because polymer biodegradation is caused by naturally occurring microorganisms and secreted polymer-degrading enzymes that basically recognize naturally occurring polymer structures and their analogs. Thus, tPAA biodegradation by isolated microorganisms or purified enzymes should be examined in order to elucidate its reaction mechanism. To this end, we have isolated tPAA-degrading bacteria and enzymes from the natural environment and investigated their tPAA degradation behavior (Tabata et al. 1999, 2000, 2001; Hiraishi et al. 2003a, b, 2004, 2009; Hiraishi and Maeda 2011). We previously isolated two tPAA-degrading bacteria, *Sphingomonas* sp. KT-1 and *Pedobacter* sp. KP-2, from river water and purified two types of enzyme that may participate in tPAA biodegradation. Recently, we have characterized PAA hydrolase-1 (PahZ1_KP-2_) from *Pedobacter* sp. KP-2 and examined its tPAA-degrading behavior. Nuclear magnetic resonance (NMR) analysis suggests that the enzyme selectively, but not completely, cleaves the amide bonds between β-Asp units in tPAA. As stated before, as the tPAA molecule contains equivalent numbers of d- and l-Asp units, unnaturally occurring sequences (d–d, d–l, l–d) formed by these units in addition to the l–l sequence may be responsible for the incomplete hydrolysis of the β–β amide bonds in tPAA by PahZ1_KP-2_. In addition, we demonstrated the enzymatic polymerization of diethyl l-Asp to obtain β-poly(Asp) (β-PAA) by taking advantage of the substrate recognition by PahZ1_KP-2_. The synthesized β-PAA had a relatively low molecular weight in the range of 750–2,500 (MALDI-TOF MS analysis) and the reaction needs a relatively large amount of PahZ1_KP-2_ (Hiraishi et al. 2011). This low activity of PahZ1_KP-2_ is also probably attributed to its substrate stereoselectivity. In the present study, to clarify the substrate stereoselectivity of PahZ1_KP-2_, we conducted the enzymatic hydrolysis of β-tri(Asp)s having well-defined sequences of d- and l-Asp units using PahZ1_KP-2_ and examined the substrate-binding site of the enzyme.

## Materials and methods

### Materials, bacterial strains, culture conditions, plasmid, and genetic procedures

The β-tri(Asp)s with all possible combinations of l- and d-Asp units were designed, and the synthetic tri(Asp)s and di(Asp)s were purchased from Medical & Biological Laboratories. All of the other chemicals used were of biochemical grade or the highest purity, and were used without further purification. *Escherichia coli* JM109 (Takara) and BL21(DE3) (Novagen) were used as the cloning and expression hosts, respectively. *E. coli* was grown in Luria–Bertani (LB) broth (1% Bacto trypton, 0.5% Bacto yeast extract, and 0.5% NaCl, pH 7.0) containing 100 µg/mL ampicillin. pET-15b (Novagen) was used as the expression vector for His-tagged PahZ1_KP-2_. All of the DNA-modifying enzymes for genetic engineering were purchased from Takara and Toyobo. The enzymes were used in accordance with the supplier’s instructions. Gene manipulations were performed in accordance with standard procedures (Sambrook and Russell 2001).

### Expression and purification of PahZ1_KP-2_

PahZ1_KP-2_ was expressed using the recombinant *E. coli* BL21(DE3) harboring pPAA1KP-2, in which the enzyme was designed as a His-tagged fusion protein, as was reported in our previous work (Hiraishi et al. 2009). PahZ1_KP-2_ was purified from the soluble fraction of the recombinant *E. coli* with a cOmplete His-Tag Purification Column (Roche Applied Science). After the sample solution was applied, the column was washed with buffer A (50 mM NaH_2_PO_4_, 300 mM NaCl, pH 8.0). The enzyme was eluted with buffer B (50 mM NaH_2_PO_4_, 300 mM NaCl, and 150 mM imidazole, pH 7.4). The enzyme fractions were collected, concentrated, and exchanged with 10 mM phosphate buffer (pH 7.0). The purity and concentration of the purified PahZ1_KP-2_ were determined by SDS-PAGE and the Bradford method, respectively (Laemmli 1970; Bradford 1976).

### Gel permeation chromatography (GPC) analysis of products of hydrolysis of tri(Asp)s and di(Asp)s by PahZ1_KP-2_

The hydrolysis of synthetic tri(Asp)s by PahZ1_KP-2_ was investigated for one α-tri(Asp) and eight β-tri(Asp)s as follows. The tri(Asp) substrate (1 mM) was incubated with 1 µM or 200 nM PahZ1_KP-2_ at 37°C in 10 mM phosphate buffer (pH 7.0) For di(Asp)s [(β-l-Asp)-(l-Asp) and (β-l-Asp)-(d-Asp)], the dimers (1 mM) were hydrolyzed at 10 µM PahZ1_KP-2_. The mixture after the enzymatic reaction was heated at 100°C for 10 min. After centrifugation of the mixture at 2,200×*g* and 4°C for 10 min, the resultant supernatant was subjected to GPC using Superdex peptide PE7.5/300 (GE Healthcare). Phosphate-buffered saline (PBS) was used as the eluent at a rate of 0.25 mL/min. The hydrolyzed products were detected at 214 nm using a UV spectrophotometer.

### Detection of l-Asp generated during β-tri(Asp) hydrolysis by PahZ1_KP-2_

l-Asp generated during β-tri(Asp) hydrolysis by PahZ1_KP-2_ was determined as follows. The hydrolysis was conducted under the above-mentioned conditions except the enzyme concentration (10 µM) and reaction temperature (30°C). After the reaction, the amount of l-Asp generated during the hydrolysis was determined under the following conditions. The reaction mixture (50 µL) was added to a solution containing 7.5 µg/mL glutamic oxaloacetic transaminase (GOT), 6.6 µg/mL malate dehydrogenase (MDH), 4 mM α-ketoglutarate, and 0.4 mM NADH in 50 µL of 10 mM phosphate buffer (pH 7.0), and absorbance was measured at 340 nm. l-Asp generated during the hydrolysis was calculated from the disappearance rate of the absorbance at 340 nm derived from the decrease in the concentration of NADH, and sodium l-Asp was used as standard.

## Results

### Enzymatic hydrolysis of (α-l-Asp)-(α-l-Asp)-(l-Asp) and (β-l-Asp)-(β-l-Asp)-(l-Asp) by PahZ1_KP-2_

**Figure 1:**
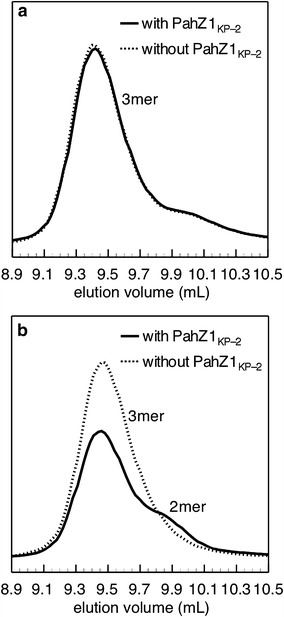
GPC curves of products during the enzymatic hydrolysis of (α-l-Asp)-(α-l-Asp)-(l-Asp) (**a**) and LLL (**b**) with or without PahZ1_KP-2_. Each substrate (1 mM) was incubated with or without the enzyme (1 µM) in 10 mM phosphate buffer (pH 7.0) at 37°C for 24 h.

To examine the effects of α- and β-amide linkages on the hydrolysis by PahZ1_KP-2_, the products of the enzymatic hydrolysis of (α-l-Asp)-(α-l-Asp)-(l-Asp) and (β-l-Asp)-(β-l-Asp)-(l-Asp) (LLL) were analyzed by GPC. Figure 1 shows the elution profiles of (α-l-Asp)-(α-l-Asp)-(l-Asp) and LLL treated with or without PahZ1_KP-2_ for 24 h. In the figure, the Asp monomer was hardly detected because of its low absorbance. The elution profile of (α-l-Asp)-(α-l-Asp)-(l-Asp) treated with PahZ1_KP-2_ was identical to that treated without PahZ1_KP-2_ (Figure 1a). In contrast, when LLL was used as a substrate, its peak area decreased and a new peak of (β-l-Asp)-(l-Asp) appeared (Figure 1b).

### GPC analysis of products on hydrolysis of β-tri(Asp)s by PahZ1_KP-2_

**Figure 2:**
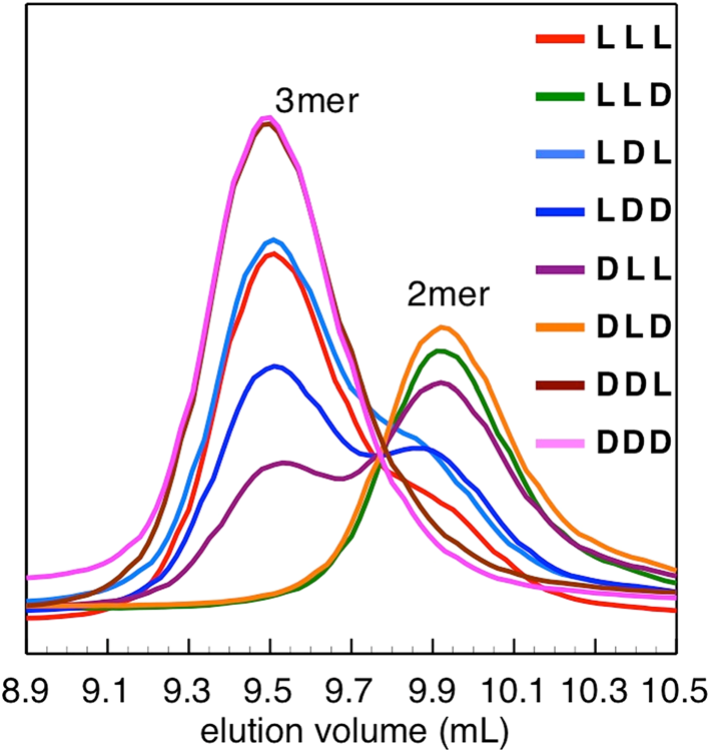
GPC curves of products of the enzymatic hydrolysis of β-tri(Asp)s by PahZ1_KP-2_. Each substrate (1 mM) was incubated with the enzyme (1 µM) in 10 mM phosphate buffer (pH 7.0) at 37°C for 24 h. LLL, LLD, LDL, LDD, DLL, DLD, DDL, and DDD are indicated in *red,* *green*, *sky blue*, *blue*, *purple*, *orange*, *brown*, and *pink*, respectively.

To elucidate the importance of the sequences of l- and d-Asp units in β-tri(Asp)s in enzymatic cleavage, eight β-tri(Asp)s with all possible combinations of l- and d-Asp units were designed and enzymatically hydrolyzed by 1 µM PahZ1_KP-2_. The GPC profiles of the hydrolytic products of β-tri(Asp)s after 24 h are shown in Figure 2. After incubation for 24 h, the degree of hydrolysis of β-tri(Asp)s was in the order of LLD = DLD ≫ DLL > LDD > LLL = LDL ≫ DDL = DDD. When DDL and DDD were used as substrates, only the peaks of the trimers were observed. In contrast, when LLD and DLD were treated with PahZ1_KP-2_ for 24 h, their peaks completely disappeared and new peaks of Asp dimers were concomitantly generated. Moreover, when the products of LLD and DLD after hydrolysis for 1 and 3 h were analyzed, the peaks of the trimers completely disappeared and those of the dimers appeared (data not shown). Even when the reaction was carried out at a low concentration of PahZ1_KP-2_ (200 nM), the peaks of the trimers completely disappeared and those of the dimers appeared after incubation for only 1 h (data not shown). In addition, the peak areas of the dimers after treatment with 200 nM PahZ1_KP-2_ for 1 h were identical to those after treatment with 1 µM enzyme for 24 h.

### Enzymatic hydrolysis of (β-l-Asp)-(l-Asp) and (β-l-Asp)-(d-Asp) by PahZ1_KP-2_

**Figure 3:**
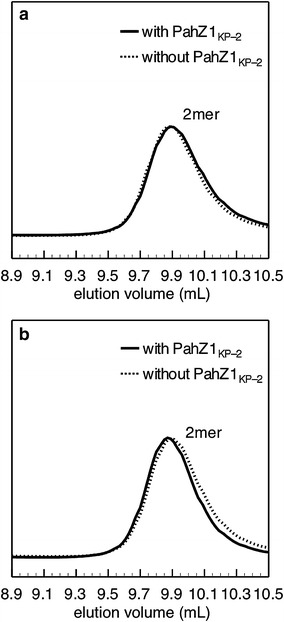
GPC curves of the reaction mixture of (β-l-Asp)-(l-Asp) (**a**) and (β-l-Asp)-(d-Asp) (**b**) treated with or without PahZ1_KP-2_. Each substrate (1 mM) was incubated with or without the enzyme (10 µM) in 10 mM phosphate buffer (pH 7.0) at 37°C for 24 h.

To investigate the minimum units for cleavage to occur, the hydrolysis of (β-l-Asp)-(l-Asp) and (β-l-Asp)-(d-Asp) dimers by PahZ1_KP-2_ was conducted as described in “Materials and methods” section. Figure 3 shows the GPC profiles of the reaction mixture treated with or without PahZ1_KP-2_ for 24 h. For both dimers, the peak areas of the dimers treated with the enzyme were identical to those treated without the enzyme.

### l-Asp generation during enzymatic hydrolysis of β-tri(Asp)s

**Figure 4:**
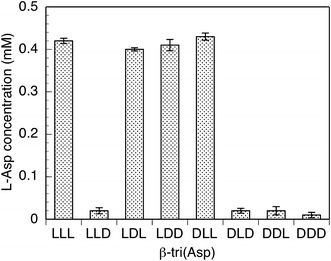
l-Asp generation during β-tri(Asp) hydrolysis by PahZ1_KP-2_. Each substrate (1 mM) was incubated with the enzyme (10 µM) in 10 mM phosphate buffer (pH 7.0) at 30°C for 24 h.

To identify the part in β-tri(Asp)s hydrolyzed by PahZ1_KP-2_, l-Asp generated during the enzymatic hydrolysis was measured. The results are shown in Figure 4. When LLD, DLD, DDL, and DDD were used, very little l-Asp was detected. However, this is most likely due to the auto-oxidation of NADH to NAD during the l-Asp quantification assay. When DDL and DDD were treated with PahZ1_KP-2_, no l-Asp was generated on hydrolysis. In the case of LLL, LDL, LDD, and DLL, l-Asp was generated. In contrast, when LLD and DLD were used as substrates, no l-Asp was detected despite their complete hydrolysis by PahZ1_KP-2_, as shown in Figure 2.

## Discussion

In this study, the substrate stereoselectivity of PahZ1_KP-2_ was investigated to reveal the substrate recognition mechanism of PahZ1_KP-2_ in tPAA hydrolysis. First, the enzymatic hydrolysis of α-tri(l-Asp) and β-tri(l-Asp) by PahZ1_KP-2_ was carried out to examine the effects of α- and β-amide linkages on the hydrolysis. The results indicate that PahZ1_KP-2_ can hydrolyze the β-amide bond, but not the α-amide bond, which are consistent with the results of our previous NMR study of the products of tPAA hydrolysis by PahZ1_KP-2_ (Hiraishi et al. 2009).

To determine the effects of the sequences of l- and d-Asp units on the β-tri(Asp) hydrolysis by PahZ1_KP-2_, we conducted GPC analysis of the enzymatic hydrolysis of eight β-tri(Asp)s with all possible combinations of l- and d-Asp units by PahZ1_KP-2_. When DDL and DDD were used as substrates, no enzymatic hydrolysis of the trimers was observed, indicating that PahZ1_KP-2_ cannot recognize (d-Asp)-(l-Asp) and (d-Asp)-(d-Asp) sequences in the substrates. Although LLL was hydrolyzed by PahZ1_KP-2_, its hydrolysis rate was not very high, demonstrating that the (l-Asp)-(l-Asp) sequence can be recognized by PahZ1_KP-2_. In the case of LLD and DLD, these trimers were quickly and completely hydrolyzed to generate the dimers. As the (l-Asp)-(l-Asp) sequence was cleaved with low efficiency and the (d-Asp)-(l-Asp) sequence was not hydrolyzable by PahZ1_KP-2_, the (l-Asp)-(d-Asp) sequence may be the most acceptable sequence for PahZ1_KP-2_. The present findings are consistent with those of our previous study (Hiraishi et al. 2009), and the substrate stereoselectivity of PahZ1_KP-2_ is probably responsible for its specific but incomplete cleavage of the amide bonds between β-Asp units in tPAA.

In addition, the GPC elution profiles of LLD and DLD treated with 200 nM PahZ1_KP-2_ for 1 h were identical to those treated with 1 µM enzyme for 24 h, respectively. The results illustrate that the possible Asp dimers [(β-l-Asp)-(l-Asp), (β-l-Asp)-(d-Asp), and (β-d-Asp)-(l-Asp)] generated upon the hydrolysis of LLD and DLD were not acceptable as substrates for PahZ1_KP-2_. To determine the minimum units for cleavage to occur, the hydrolysis of (β-l-Asp)-(l-Asp) and (β-l-Asp)-(d-Asp) dimers was carried out at 10 µM PahZ1_KP-2_. GPC analysis showed that the peak areas of (β-l-Asp)-(l-Asp) and (β-l-Asp)-(d-Asp) treated with the enzyme were identical to those treated without the enzyme, respectively. This means that no enzymatic hydrolysis of both dimers proceeds and the Asp trimers are the minimum units for the hydrolysis by PahZ1_KP-2_.

When DDL was treated with PahZ1_KP-2_, no l-Asp was generated on hydrolysis, suggesting that the (d-Asp)-(l-Asp) sequence was not digested by PahZ1_KP-2_, consistent with the results shown in Figure 2. In contrast, l-Asp generation was observed during the hydrolysis of LLL, LDD, and DLL. This indicated that the amide bonds of the (l-Asp)-(l-Asp) and (l-Asp)-(d-Asp) sequences in β-tri(Asp)s were truncated by the enzyme. During the hydrolysis of LDL, as the (d-Asp)-(l-Asp) sequence was not an acceptable substrate for PahZ1_KP-2_ as stated above, l-Asp was generated by cleaving the amide bond between (l-Asp)-(d-Asp) in LDL. In the case of LLD and DLD hydrolysis, no l-Asp was detected despite their complete hydrolysis by PahZ1_KP-2_, as shown in Figure 2, indicating that only the amide bond between (β-l-Asp)-(d-Asp) was cleaved by PahZ1_KP-2_ to generate d-Asp. Taken together, the results attest that PahZ1_KP-2_ cleaves the amide bond at the carboxyl part of the β-l-Asp unit in stereoisomeric β-tri(Asp)s to generate (l-Asp)-(l-Asp), (d-Asp)-(l-Asp), and (d-Asp)-(d-Asp) dimers.

**Figure 5:**
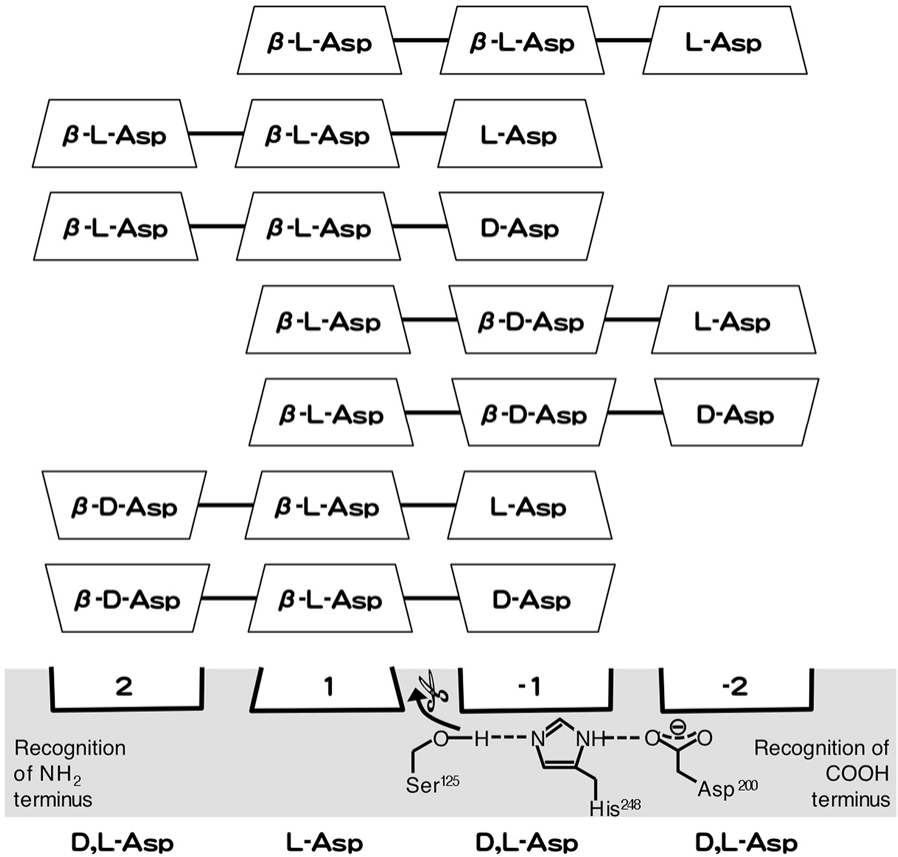
Schematic model of substrate recognition site of PahZ1_KP-2_.

On the basis of the present findings, we propose the following model of substrate binding of PahZ1_KP-2_ (Figure 5). PahZ1_KP-2_ cleaves the β-amide bond between the (l-Asp)-(l-Asp) sequence as well as that between the (l-Asp)-(d-Asp) sequence when the Asp unit attaches to the N-terminus or the C-terminus of those sequences to form Asp trimers. In addition, the optical isomerism of the attached Asp unit affects the hydrolysis rate of the trimers (Figure 2). These indicate that the substrate-binding site of PahZ1_KP-2_ is composed of at least four subsites (subsites 2, 1, −1, and −2). When three of the four subsites are occupied by Asp units, amide bond cleavage occurs between subsites 1 and −1. Subsite 1 can recognize only the l-Asp unit, whereas the other subsites can accept both the l- and d-Asp units. Among the dimer sequences, the (l-Asp)-(d-Asp) sequence is the most acceptable as the two central subsites.

Except for the tPAA-hydrolyzing enzymes, five β-peptidyl aminopeptidases (three BapA enzymes, one BapF enzyme, and one DmpA enzyme) are known as the enzymes hydrolyzing short β-peptides and β-amino-acid-containing peptides (Geueke and Kohler 2007; Heck et al. 2009; Fuchs et al. 2011). Their general reaction involves the N-terminal cleavage of β-amino acid units from oligopeptides, amides, and esters via an exo-type process. Geueke et al. (2006) demonstrated that BapA enzymes from 3-2W4 and Y2 strains can accept β-dipeptides as substrates as well as β-tripeptides. They also investigated the hydrolysis of mixed β/α-tripeptides and suggested that a peptide required a β-amino acid unit at its N-terminus in order to be a substrate of BapA. The effects of the optical isomerism of the N-terminal unit in β-tripeptides on the hydrolysis by BapA enzymes indicated that the enzymes preferred the peptide with the l-configuration of the N-terminal unit as a substrate. Heck et al. (2006) examined the substrate specificity of DmpA and demonstrated that DmpA can cleave α-peptides in addition to β-peptides, but the hydrolysis rate of α-peptides was lower than that of β-peptides. According to the MEROPS database (Rawlings et al. 2010), these enzymes are classified into the peptidase family P1 containing aminopeptidases and self-processing proteins. From structural features of DmpA and BapA, they belong to the N-terminal nucleophile (Ntn) hydrolases that self-activate to produce two subunits (α and β-subunits) for forming the active enzymes (Merz et al. 2012).

In contrast to these β-peptidyl aminopeptidases, PahZ1_KP-2_ degrades the β-amide bonds in tPAA polymer via an endo-type process (Hiraishi et al. 2009) and cleaved both the N- and C-terminal Asp units of β-tri(Asp)s depending on the position of the β-l-Asp unit in the molecule, as stated above. In addition, PahZ1_KP-2_ accepts β-tri(Asp)s as substrates, but not β-di(Asp)s. From the MEROPS database, PahZ1_KP-2_ is classified into the peptidase family S9, which contains various sets of Ser-dependent peptidases and generally has an α/β-hydrolase fold in their structure. From the viewpoint of structural features, PahZ1_KP-2_ exists in a monomer form to exert its hydrolysis activity (Hiraishi et al. 2009).

Previous sequence analyses demonstrated that the amino acid sequence of PahZ1_KP-2_ has a high similarity to that of PAA hydrolase-1 from *Sphingomonas* sp. KT-1 (PahZ1_KT-1_) showing similarity to poly(*R*-3-hydroxybutyrate) (PHB) depolymerases (Hiraishi et al. 2003a, 2009). PHB depolymerases are monomer enzymes having the α/β-hydrolase fold and cleave the β-ester bonds in PHB via an endo-type process (Jendrossek and Handrick 2002; Hisano et al. 2006; Wakadkar et al. 2010). Earlier studies using oligo(3-hydroxybutyrate)s having well-defined sequences provided general information about the substrate-recognition site of PHB depolymerases as follows: (1) the active site contains four subsites (2, 1, −1, and −2), three of which are occupied by monomer units for cleavage to occur; (2) for the hydrolysis to proceed, subsite 1 and −1 should be occupied by the *R*-3-hydroxybutyrate unit, whereas the other two subsites may accept both *R*- and *S*-3-hydroxybutyrate units (Bachmann and Seebach 1999; Hiraishi et al. 2000; Scherer et al. 2000). Thus, the substrate recognition mechanism of PHB depolymerases seems to be similar to that of PahZ1_KP-2_, although the mechanism of recognition of optical isomers at subsite 1 differs from each other. On the basis of these functional and structural findings, it is assumed that PahZ1_KP-2_ shares a common ancestor with PHB depolymerases rather than β-peptidyl aminopeptidases.

## Abbreviations

LLL: (β-l-Asp)-(β-l-Asp)-(l-Asp)
LLD: (β-l-Asp)-(β-l-Asp)-(d-Asp)
LDL: (β-l-Asp)-(β-d-Asp)-(l-Asp)
LDD: (β-l-Asp)-(β-d-Asp)-(d-Asp)
DLL: (β-d-Asp)-(β-l-Asp)-(l-Asp)
DLD: (β-d-Asp)-(β-l-Asp)-(d-Asp)
DDL: (β-d-Asp)-(β-d-Asp)-(l-Asp)
DDD: (β-d-Asp)-(β-d-Asp)-(d-Asp)
